# The quality and safety of establishing Ireland’s first robotic adrenalectomy program: a single centre experience

**DOI:** 10.1007/s00423-026-04083-6

**Published:** 2026-05-21

**Authors:** Mohammed Al Azzawi, Antonina Tecancenco, Lorcan Lalor, Fiachra McHugh, Taqi Al Zubaidi, Oluwatofarati J. Sokan, Jelizaveta Cvetkova, Gordon Daly, Michael W. O’Reilly, Mark Sherlock, Neal Dugal, Arnold D. K. Hill

**Affiliations:** 1 Department of General Surgery, Beaumont Hospital, Beaumont, Dublin 9, Ireland; 2 Department of Surgery, Royal College of Surgeons in Ireland, Dublin 2, Ireland; 3 Department of Endocrinology, Beaumont Hospital, Dublin 9, Beaumont, Ireland

**Keywords:** Adrenal tumours, Minimally invasive surgery, Robotic adrenalectomy, Robotic-assisted surgery, Phaeochromocytoma

## Abstract

**Background:**

Robotic adrenalectomy offers several technical advantages over conventional laparoscopy, particularly for lesions located adjacent to major vascular structures. However, data regarding the establishment of new robotic adrenalectomy programmes remains limited. This study aimed to evaluate the safety, feasibility, and early outcomes of implementing the first robotic adrenalectomy programme in the Republic of Ireland.

**Methods:**

A retrospective review of the first 50 consecutive patients who underwent robotic adrenalectomy between November 2021 and December 2024 at a tertiary endocrine surgery referral centre was performed. Clinical, pathological, and perioperative outcomes were analysed. Continuous variables were compared using Student’s t-test or Mann–Whitney U test, and multivariable linear regression was used to assess factors associated with operative duration.

**Results:**

The mean age was 58.3 years, and 72% of patients were male. Median body mass index was 29.1 kg/m². The most common indications for surgery were phaeochromocytoma (32%), Cushing’s syndrome (22%), and non-functioning lesions (18%). Median operative duration was 120 min (range 80–210). Conversion to open surgery occurred in 1 patient (2%), and intraoperative complications occurred in 3 patients (6%). Mean length of stay was 4.0 days. Postoperative morbidity was low, with four Clavien–Dindo grade II complications and one grade IIIa complication. There were no 30-day mortalities, reoperations, or readmissions. Complete (R0) resection was achieved in 94% of cases. Patients with larger tumours had longer operative duration and length of stay, whereas elevated BMI was not associated with increased operative duration.

**Conclusions:**

Robotic adrenalectomy is a safe and feasible minimally invasive approach that can be successfully implemented within a tertiary referral endocrine surgery centre, with low morbidity, low conversion rates, and acceptable pathological outcomes.

## Background

The adrenal gland plays a central role in the endocrine system, regulating physiological functions by releasing hormones such as cortisol, aldosterone, and adrenaline [1, 2]. These hormones play a crucial role in maintaining water and electrolyte balance, supporting the immune system, and regulating metabolism [1, 2]. Dysregulation of these hormones results in a variety of adrenal-related conditions, such as Cushing’s syndrome, Conn’s syndrome, phaeochromocytoma, and others [3–5]. Adrenalectomy remains the gold standard for the management of most adrenal conditions, and laparoscopic resection has become the most commonly adopted approach since its introduction by Gagner et al. in 1992 [6, 7]. Laparoscopic adrenalectomy gained popularity over the years in benign and malignant adrenal conditions due to a lower complication rate, shorter hospitalisation and faster recovery than open surgery [8, 9]. The size of the adrenal lesion has been controversial in laparoscopic surgery; however, over time, larger benign lesions were operated on laparoscopically [10, 11]. The SAGES 2026 guidelines recommend that laparoscopic resection should be attempted for larger tumours (less than 7.5 cm) with no evidence of malignancy preoperatively or intraoperatively [12]. Laparoscopic resection remains challenging owing to the rigidity of instruments and the gland’s anatomical location. Classically, larger tumours are excised using open adrenalectomy due to anatomical challenges and potential intraoperative complications [7, 8, 9]. Robotic surgery, however, has changed this narrative [10].

Robotic surgery has gained popularity in recent years and has been shown to be superior to laparoscopic surgery in several respects [13]. Instrument dexterity and flexibility, enhanced depth of perception, three-dimensional vision, and improved ergonomics in robotic surgery encouraged its adoption [10]. Moreover, robotic surgery enables more extensive, meticulous dissection, thereby improving nodal yield and potentially enhancing oncological outcomes in cancer surgery compared with laparoscopy [14, 15]. Superior instrumentation and enhanced ergonomics enable precise resection of the adrenal gland despite its challenging anatomical position adjacent to major vessels, necessitating meticulous dissection [16].

Despite increasing adoption of robotic adrenalectomy, there remains limited data on the safe implementation of new robotic programmes at a national level, particularly within publicly funded healthcare systems. In this study, we report on the safety and feasibility of establishing the first robotic adrenalectomy program in the Republic of Ireland. We present a single-centre experience of robotic adrenalectomy by reporting on the clinical, pathological and perioperative outcomes of our patient cohort.

## Methodology

This is a retrospective review of the first 50 patients who underwent robotic adrenalectomy at a tertiary university hospital. Two consultant surgeons, both experienced in laparoscopic adrenalectomy, performed the first fifty robotic adrenalectomies at a tertiary endocrine surgery referral centre between November 2021 and December 2024. The Institutional Ethics Review Board approved proceeding with the project, and data collection and verification were finalised by April 2025.

An adrenalectomy database was created and prospectively maintained, including patients’ basic demographics, clinical and pathological characteristics, and perioperative outcomes. Patient fitness was assessed using the American Society of Anaesthesiologists (ASA) grading [17]. Post-operative complications were classified according to the Clavien-Dindo classification [18].

### Setting up the robotic adrenalectomy program – institutional management protocols

The program was launched in November 2021, and robotic adrenalectomy was performed using the Da Vinci X and Da Vinci Xi platforms. Patients with an incidental adrenal lesion are referred to the endocrine department for biochemical and radiological assessments. As a standard initial imaging, patients undergo a contrast-enhanced Computed Tomography (CT) of the adrenal glands. Other radiological investigations include magnetic resonance imaging (MRI) of the adrenals, metaiodobenzylguanidine (MIBG) scintigraphy, and positron emission tomography (PET). The imaging modality was selected based on the suspected underlying pathology. The biochemical workup is tailored to the patient’s condition and the underlying pathology. Urinary catecholamines, plasma metanephrines, normetanephrines, and 3-methoxytyramine are measured to diagnose phaeochromocytoma. Plasma renin and aldosterone levels are requested to investigate for Conn’s syndrome, and urinary/plasma cortisol levels and Synacthen test are performed to assess for Cushing’s disease.

Patients underwent familial genetic investigations, where applicable. Genetic testing was performed when suspicion of an underlying genetic mutation arose. All patients were discussed at the Endocrinology Multidisciplinary Team Meeting to determine the course of treatment. All patients underwent a preoperative assessment to ensure surgical suitability. Phaeochromocytoma patients were admitted five days preoperatively for alpha-blockade, followed by beta-blockade by the endocrinology team. Patients recover in the Intensive Care Unit, if warranted, and are typically discharged within 3–4 days postoperatively. Patients are followed up in the outpatient department 4 weeks after the final histopathology results are available.

### Robotic adrenalectomy – the surgical technique

**Fig. 1:**
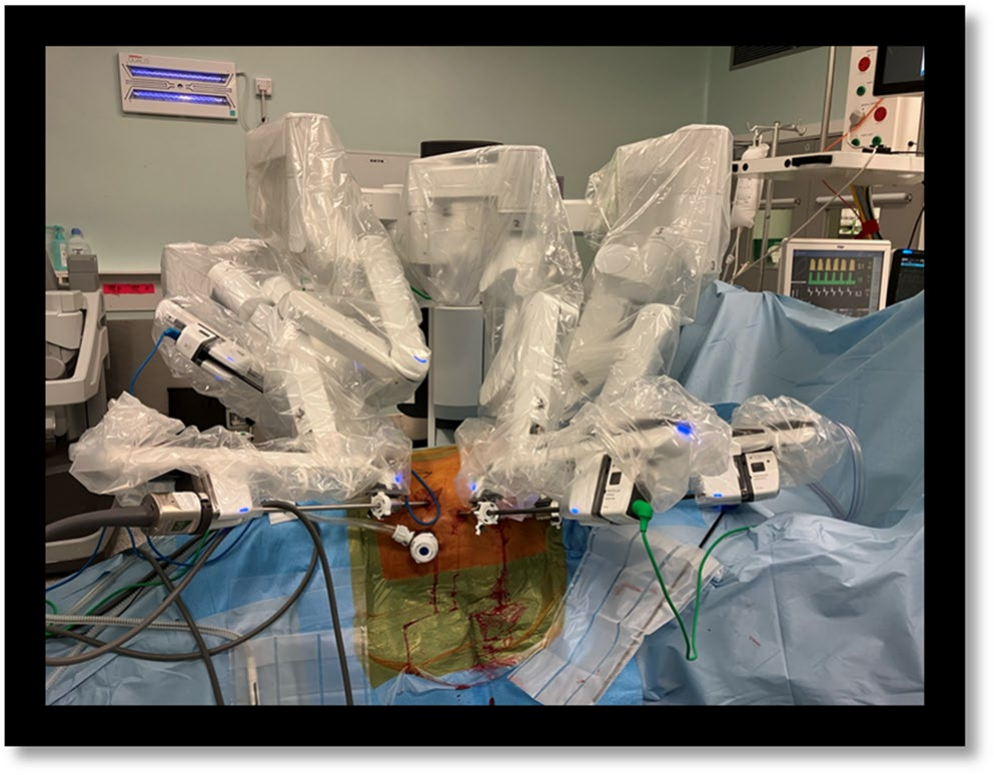
Patient positioning and ports placement: the figure demonstrates the standard patient positioning and docking of the Da Vinci X platform during a robotic adrenalectomy

Robotic adrenalectomy is typically performed using a transperitoneal lateral approach. The patient is positioned in a full or modified lateral decubitus position with the affected lesion side upwards to maximise exposure of the upper retroperitoneum. Pneumoperitoneum is achieved via the Veress needle, and the robotic ports and an assistant port are inserted under direct visualisation in a configuration that allows adequate triangulation toward the adrenal gland and minimises arm collision. The ports are placed in a straight line from the camera port, with one port cephalad to it and two ports caudal to it. An assistant port is inserted adjacent to the umbilicus, after which the robotic platform is docked. The ports are placed in a straight line from the robotic port, with one on the left and two on the right. An assistant port is inserted superior to the umbilicus. Figure 1 illustrates the standard positioning and port placements.

**Fig. 2:**
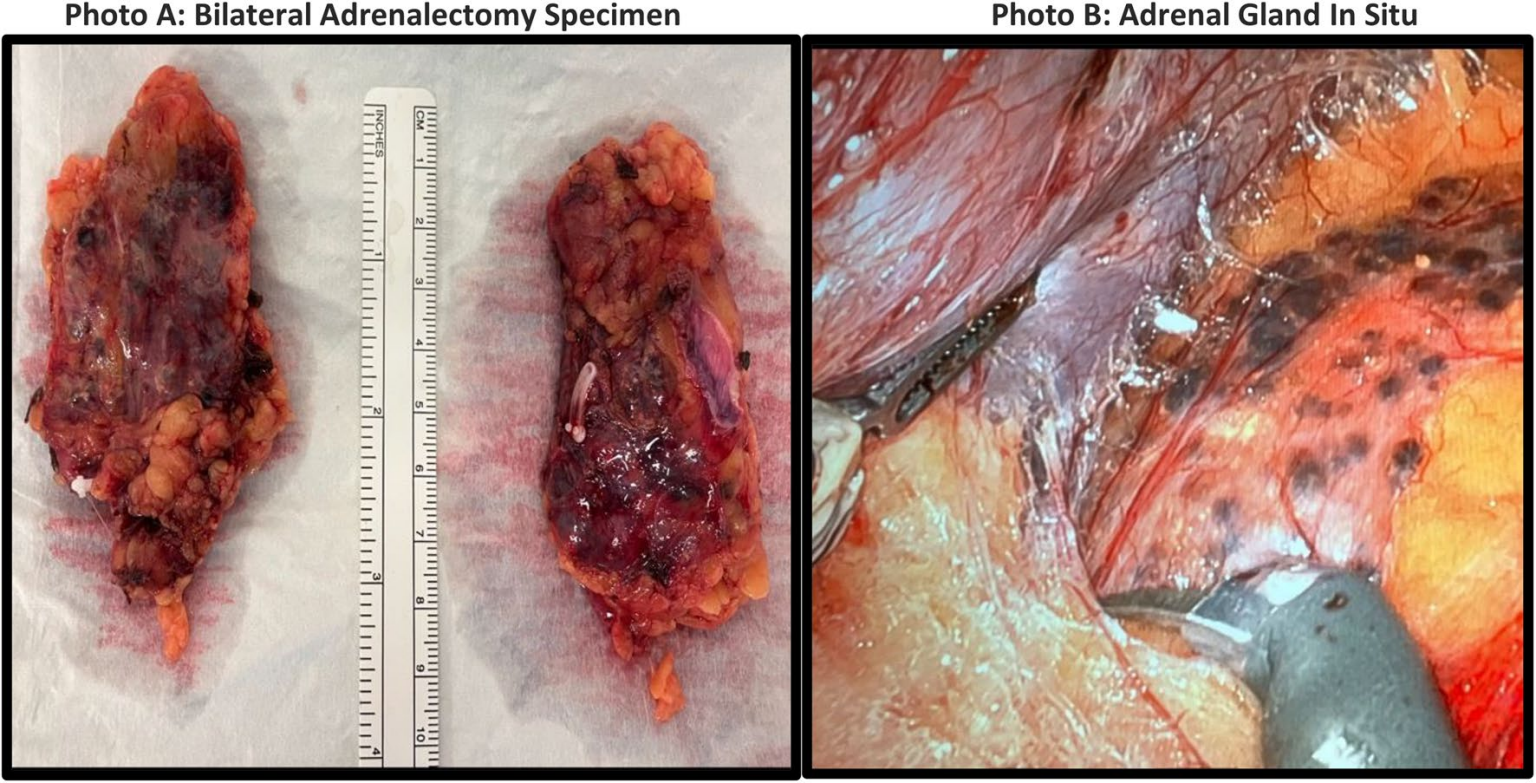
Bilateral adrenalectomy in a patient with cushing’s disease: this is the case of a 17-year-old male who underwent robotic bilateral adrenalectomy for Cushing’s disease. Photo **A** illustrates the specimen of both adrenal glands, and Photo **B** is an intracorporeal photograph of the adrenal gland in situ with PPNAD pigmentation

Exposure is obtained according to the side of the surgery. On the right, the liver is retracted superiorly with a Prograsp instrument controlled by the fourth robotic arm exposing the retrohepatic space and inferior vena cava. Whereas on the left, the splenic flexure is mobilised, and the spleen and tail of the pancreas are reflected medially to expose the adrenal region. The upper pole of the kidney, surrounding perinephric fat, and major vascular structures are identified to define the adrenal gland. Early identification and control of the adrenal vein are key steps; it is dissected and divided near the inferior vena cava on the right and at the junction of the left renal vein on the left using two hemolock clips. The gland is then carefully mobilised using monopolar diathermy with scissors and the vessel sealer, with meticulous control of small arterial and venous branches while preserving the capsule and protecting adjacent organs. Once completely freed from its superior, lateral, medial, and inferior attachments, the specimen is placed in an EndoCatch retrieval bag and extracted through the umbilical assistant port site. The operative field is inspected for haemostasis, and haemostatic agents are sprayed over the operative site, and a Transversus Abdominus Plane block is given for pain management. Following undocking, the ports are removed under direct vision, and the wounds are closed in standard fashion. Figure 2 shows the histopathology specimen from a patient undergoing bilateral adrenalectomy for Cushing’s disease.

### Statistical analysis

Descriptive statistics were performed for clinical and pathological characteristics. Continuous variables were described using medians and means along with their corresponding standard deviations and were compared using the unpaired Student’s t-test and the Mann-Whitney. Medians are described with corresponding ranges and means with standard deviations (SD). A multivariate linear regression was performed to assess the relationship between the procedure duration and the explanatory variables: ASA grade, tumour size, BMI (kg/m²), and age. Data were checked for multicollinearity with the Belsley-Kuh-Welsch technique. Heteroskedasticity and normality of residuals were assessed, respectively, by the Breusch-Pagan test and the Shapiro-Wilk test. A p-value < 0.05 was considered statistically significant for all analyses. Patients with missing data were excluded from analysis. Statistical analysis was performed using SPSS Statistics for Windows, version 29 (SPSS Inc., Chicago, Ill., USA), and GraphPad Prism version 8.4.2 (GraphPad Software LLC, San Diego, California, USA).

## Results

### Basic demographics

The mean age of participants was 58.3 (SD 14.7); 72% (*n* = 36) were men. Of these, 74% were ASA III (*n* = 37) and 26% were ASA II (*n* = 13). The median BMI was 29.1 (19–72). Most patients underwent contrast-enhanced CT of the adrenal glands, whereas additional imaging modalities included adrenal MRI and MIBG scintigraphy in selected cases. The most common diagnoses in our cohort were phaeochromocytoma (*n* = 16, 32%), Cushing’s syndrome (*n* = 11, 22%), and non-functioning lesions (*n* = 9, 18%). In total, four patients were diagnosed with distinct genetic conditions: Li-Fraumeni Syndrome (due to a TP53 gene mutation), Neurofibromatosis type II, Klippel-Trenaunay syndrome, and Succinate Dehydrogenase Complex Iron-Sulfur Subunit B (SDHB) gene mutation. The basic demographics are presented in Table 1.

**Table 1:** Basic demographics of the robotic adrenalectomy patients: basic demographics include sex, age, ASA score, BMI, and clinical diagnosis. Outcomes with * have been reported using mean and standard deviation

Basic Demographics of The Robotic Adrenalectomy Cohort (*n = *50)		
Sex	Male	36 (72%)
	Female	14 (28%)
Age	Mean	58.3 (SD 14.7)
ASA Score	ASA II	13 (26%)
	ASA III	37 (74%)
BMI*	Median	29.1 ( Range 19-72)
Pre-operative Diagnosis	Phaeochromocytoma	16 (32%)
	Cushing’s Syndrome	11 (22%)
	Conn’s Syndrome	8 (16%)
	Non-functioning lesions	9 (18%)
	Mixed Pathology	4 (8%)
	Paraganglioma	2 (4%)

### Clinical and pathological outcomes

Out of the fifty cases, 47 had unilateral adrenal lesions, two had a left para-aortic paraganglioma, and one patient underwent bilateral adrenalectomy. The median operative duration was 120 min (Range 80–210 min). Three cases had intraoperative complications, and one case was converted to open due to inadvertent injury to the small bowel during port insertion. Four patients had a planned admission to the Intensive Care Unit postoperatively for circulatory support and central venous monitoring following bilateral adrenalectomy or pheochromocytoma excision. Three cases had intraoperative complications, including an inadvertent injury to the small bowel during port insertion in a patient with multiple previous laparotomies; the small bowel injury was repaired via the retrieval incision at the end of the case. There were four cases with class II Clavien-Dindo morbidity. There was one case of class IIIa morbidity secondary to involvement of the coeliac trunk by a large neuroganglioma, necessitating elective conversion and vascular bypass from the supracoeliac aorta to the common hepatic artery, after tumour excision. The liver was successfully revascularised. There were no 90-day morbidities, readmissions, reoperations, or inpatient mortalities. The mean length of stay was 4.04 days (SD 3.86). The perioperative outcomes are illustrated in Table 2.

**Fig. 3:**
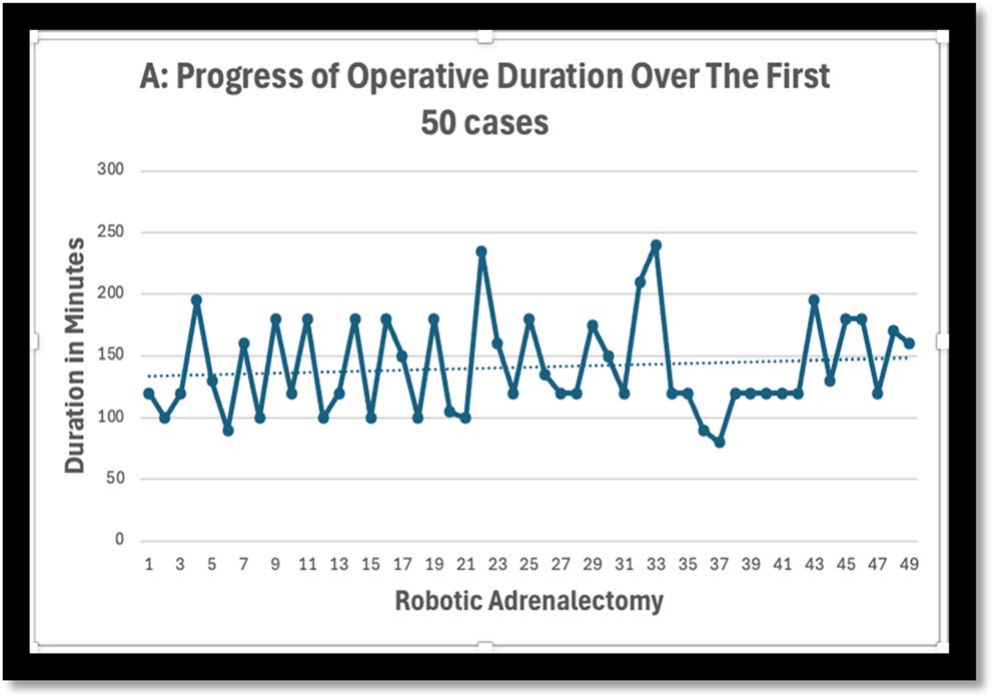
The progress of operative duration over the first 50 cases: the figure illustrates the overall progress of operative duration over the first 50 cases; one case was excluded due to conversion to open

The mean size of the lesion on radiology was 41.5 mm, the mean specimen size was 78 mm, and the mean size of the lesion on the final histology was 43.8. Regarding the final histological diagnosis, adrenal adenomas were identified in 46% of patients (*n* = 23), whereas phaeochromocytoma was identified in 32% (*n* = 16). A metastatic lesion from a renal cell carcinoma was identified in three patients, whereby complete resection was achieved. The remaining patients had adrenal hyperplasia, paraganglioma and mixed pathology. A Complete resection margin was achieved in 94% of cases (*n* = 47). Table 2 illustrates the pathological outcomes in our patients. Figure 3 shows the evolution of operative duration across the first 50 cases.

**Table 2:** Clinical and pathological outcomes of the robotic adrenalectomy cohort. The table includes clinical outcomes, such as lesion size and laterality, as well as pathological outcomes, including the final diagnosis, postoperative lesion size, and negative margin resection. Outcomes with (*) have been reported using the Mean and Standard Deviation

Clinical and Pathological Outcomes of the Robotic Adrenalectomy Cohort (n=50)		
Lesion Size on Radiology*	Mean	41.5 mm (SD 24.9)
	Median	37 mm (Range 9-120)
Side of the Lesion	Right	18
	Left	31
	Bilateral	1
Histological Diagnosis	Adrenal Adenoma	23 (46%)
	Phaeochromocytoma	16 (32%)
	Malignancy-Metastatic Lesion	3 (6%)
	Adrenal Hyperplasia.	2 (4%)
	Paraganglioma.	2 (4%)
	Others	4 (8%)
Specimen Size on Histology*	Mean	78 mm (SD 25)
	Median	80 mm (range 35-160)
Size on Tumour on Histology	Mean	43.8 mm (SD 28.1)
	Median	37 mm (range 0-115)
Complete Lesion Excision	R0	47
	R1	3
Operative Duration*	Median	120 minutes (range 80-210).
Conversion to open	1/50	2%
Intraoperative Complications	3/50	6%
Length Of Stay*	Mean	4.04 days ( SD 3.86)
	Median	3 day (Range 2-22)
Admission to ICU	4/50	8%
30 Day Morbidity (Clavien-Dindo classification)	II	4
	IIIa	1
30-Day Mortality	0/50	
90-Day Mortality	0/50	

### Comparison of clinicopathological characteristics according to BMI

When comparing clinical and pathological outcomes by BMI, patients were grouped into two categories: normal BMI (< 25, *n* = 13) and high BMI (> 25, *n* = 37). Age, gender and ASA scores were comparable between the two groups. Tumour size and operative duration were higher in the normal BMI group, while age, length of stay and specimen size were higher in the high BMI group. Table 3 summarises our findings by BMI.

**Table 3:** Comparison of clinicopathological characteristics according to BMI: patients were grouped into the normal BMI group (normal BMI (< 25, *n* = 13) and the high BMI group (>25, *n* = 37)

	BMI <=25 KG/M^2^ (*n* = 13)	BMI >25 KG/M^2^ (*n* = 37)	
BMI (Kg/M^2^)	22.78	33.5	
Age (Years)	47.8	54.2	*P* = 0.19
Male	31 % (*n* = 4)	30% (*n* = 11)	
Female	69% (*n* = 9)	70% (*n* = 26)	
ASA II	31% (*n* = 4)	24% (*N* = 9)	
ASA III	69 % (*n* = 9)	74% (*n* = 28)	
Duration of Operation (minutes)	145	136	*P* = 0.47
Length of Stay (Days)	3.41	3.86	*P* = 0.68
Specimen Size on Histology (mm)	77.42	80.83	*P* = 0.68
Tumour Size on Histology (mm)	50	37.8	*P* = 0.21
Phaeochromocytoma	6	10	
Cushing’s Syndrome	2	8	
Conn’s Syndrome	1	8	
Non-functioning lesions	4	10	
Mixed Pathology	0	1	

### Comparison of clinicopathological characteristics according to tumour size

Clinical and pathological outcomes were analysed according to the tumour size. Age, gender, and BMI were comparable between the two groups. Patients with larger tumours had longer operative duration (*p* = 0.17) and longer duration of stay (*p* = 0.08). ASA III was higher in the small tumour group in comparison to the large tumour group. Table 4 presents a summary of our findings, categorised by tumour size.

**Table 4:** Comparison of clinicopathological characteristics according to tumour size: patients were grouped into the small tumour group (Tumour < 3 cm (*n* = 18)) and the large tumour group (Tumour > 3 cm (*n* = 32)

	Tumour < 3 cm (*n* = 18)	Tumour > 3 cm (*n* = 32)	
Tumour Size (mm)	23.2	59.5	
BMI (Kg/M^2^)	32.92	29.48	*P* = 0.18
Age (Years)	50.7	53.6	*P* = 0.52
Male	8 (45%)	7 (22%)	
Female	10 (55%)	25 (78%)	
ASA II	3 (16%)	10 (31%)	
ASA III	15 (84%)	22 (69%)	
Duration of Operation (minutes)	131.4	160.5	*P* = 0.17
Length of Stay (Days)	2.77	4.7	*P* = 0.08
Specimen Size on Histology (mm)	73.89	82.52	*P* = 0.23
Phaeochromocytoma	3	13	
Cushing’s Syndrome	3	7	
Conn’s Syndrome	7	2	
Non-functioning lesions	5	9	
Mixed Pathology	0	1	

## Discussion

In this study, we report the successful implementation of a robotic adrenalectomy programme within a tertiary endocrine surgery centre, demonstrating that the transition to a robotic approach can be achieved safely without compromising perioperative or oncological outcomes. In our cohort, robotic adrenalectomy was associated with a low conversion rate, low perioperative morbidity, no mortality, and a high rate of R0 resection. The median BMI was 29.1 Kg/m^2^, with a maximum of 72 kg/m2, which is one of the benefits of adopting the robotic surgical approach. High BMI can impose technical challenges when performing laparoscopic adrenalectomy and is associated with increased perioperative complications [19]. Moreover, patients with higher BMI were found to have more extended hospital stays and larger tumours. Historically, lesions measuring 7.5 cm or more with evidence of preoperative or intraoperative malignancy would have required an open approach to achieve safe and complete resection (7, 8 [12], . Moreover, the European Society of Endocrinology clinical practice guidelines state that minimally invasive adrenalectomy can be performed for benign and malignant lesions measuring under 6 cm without local invasion [20]. A complete resection with negative margins was achieved in 94% of cases, and no conversions due to technical difficulty were observed in our cohort. Our perioperative complication rate was 6%, lower than the reported average of 9–12% [21, 22]. There were no 30-day or 90-day mortalities or readmissions in our cohort. This suggests that the robotic approach to adrenal surgery is safe and does not compromise oncological or perioperative outcomes.

Although laparoscopic adrenalectomy remains the established standard of care, robotic adrenalectomy has emerged as a valuable alternative, particularly in technically demanding cases [23, 24]. While the adoption of robotic adrenalectomy has been slow due to the steep learning curve and concerns regarding safety and oncological outcomes, previous studies have demonstrated that the technique is superior to the conventional laparoscopic resection [25]. Consistent with our findings, other retrospective studies have reported that robotic adrenalectomy is associated with favourable surgical outcomes, an enhanced operative experience, and improved instrument articulation and precision, which may facilitate safer dissection and vascular control [16, 26]. In our series, robotic adrenalectomy was successfully applied across a heterogeneous cohort, including patients with high BMI, functional tumours, and malignant pathology. Notably, BMI was not associated with increased operative duration, supporting existing evidence that robotic platforms may overcome some of the technical limitations encountered in obese patients undergoing minimally invasive adrenal surgery. Our BMI-based analysis revealed that BMI is not a risk factor for longer operative duration, consistent with prior studies [16, 27].

Our findings are consistent with prior retrospective and multicentre studies demonstrating that robotic adrenalectomy is associated with favourable outcomes and low complication rates. Parente et al. reported favourable outcomes with robotic versus laparoscopic adrenalectomy for phaeochromocytoma [28], while Kim et al. identified phaeochromocytoma as an independent risk factor for a long operative duration in their experience of robotic adrenalectomy [16]. In our cohort, operative duration remained variable, which likely reflects both case complexity and the learning curve associated with programme implementation. Importantly, no conversions due to technical difficulties were observed, suggesting the robotic platform can be adopted safely even during the early phases of institutional experiences. Cost-effectiveness remains a concern when it comes to robotic surgery; however, a recent study showed robotic adrenalectomy to be cost-effective when compared to other techniques when reserved for bigger and more challenging lesions [29].

This study should be interpreted within the merit of its limitations. This was a single tertiary referral centre experience and reflects the practice of two surgeons during the early adoption phase of a robotic adrenalectomy programme, which may limit the generalisability of the findings. The retrospective design introduces an inherent risk of selection bias and unmeasured confounding. The sample size is relatively small, which limits statistical power, particularly for subgroup analyses according to BMI and tumour size. In addition, the inclusion of the initial learning curve cases may have influenced operative duration and perioperative outcomes. Finally, although short-term clinical and pathological outcomes were favourable, longer-term follow-up data, including oncological outcomes and recurrence, were not available and should be evaluated in future studies.

## Conclusion

Robotic adrenalectomy is a safe and feasible minimally invasive approach that can be successfully implemented within a tertiary referral endocrine surgery centre. In this initial 50-case experience, the technique was associated with low conversion and complication rates, short hospital stay, and acceptable pathological and oncological outcomes. These findings support the role of robotic adrenalectomy as an effective approach in appropriately selected patients and provide a framework for the safe adoption of robotic adrenal surgery programmes.

## Data Availability

Data will be available upon request from the corresponding author.
